# 4350 From Translational to Transformational: Establishing CLIC’s Vision for a Research Education and Training Community

**DOI:** 10.1017/cts.2020.213

**Published:** 2020-07-29

**Authors:** Alfred Vitale, Russell Lackey, Melissa Trayhan, Robert White

**Affiliations:** 1 University of Rochester Medical Center; 2 Center for Leading Innovation and Collaboration (CLIC)

## Abstract

OBJECTIVES/GOALS: The new CLIC Education & Career Development Gateway aims to be a translational science workforce ecosystem for CTSAs to share learning and training resources and career opportunities. The Gateway also provides individualized assistance to identify and implement TS learning and training resources. METHODS/STUDY POPULATION: The CLIC Education & Career Development Gateway, located on the CLIC website, is an entry way to: 1) the Education Clearinghouse, a platform where CTSA Program hubs can find and share educational resources individually or as part of resource kits; 2) the Opportunities Board, which includes jobs and mini-sabbaticals from CTSA Program hubs; and 3) the Education & Training Navigator, a personalized approach to education and training requests. These approaches help empower and support a cooperative learning and training community that is inclusive and collaborative, facilitating and amplifying opportunities for the sharing of educational resources throughout the translational science workforce. RESULTS/ANTICIPATED RESULTS: Through a person-centered, direct engagement approach, the anticipated outcomes of these efforts are to promote increased collaboration across CTSA Program Hubs and partners, and the amplification of accessible, relevant existing resources. Another anticipated outcome is increased production of educational materials through the reduction of work duplication and identification of gaps in education and training resources. The Gateway also provides an opportunity to communicate the work and efforts that consortium-level special groups (working groups, special interest groups, etc.) produce. Ongoing evaluations and suggestions will help determine future improvements and functionalities. DISCUSSION/SIGNIFICANCE OF IMPACT: CLIC’s education and training ecosystem promotes education as a community space to facilitate opportunities for collaboration and partnerships, amplifying visibility of the work created by members of the CTSA community, and encouraging a transformative career trajectory for trainees and scholars.

